# Conscious Changes of Carbon Nanotubes Cytotoxicity by Manipulation with Selected Nanofactors

**DOI:** 10.1007/s12010-015-1607-1

**Published:** 2015-04-18

**Authors:** Karolina Werengowska-Ciećwierz, Marek Wiśniewski, Artur P. Terzyk, Katarzyna Roszek, Joanna Czarnecka, Paulina Bolibok, Gerhard Rychlicki

**Affiliations:** Physicochemistry of Carbon Materials Research Group, Faculty of Chemistry, Nicolaus Copernicus University in Toruń, 7 Gagarin St., 87-100 Toruń, Poland; INVEST-TECH R&D Center, 32-34 Plaska Street, 87-100 Toruń, Poland; Department of Biochemistry, Faculty of Biology and Environment Protection, Nicolaus Copernicus University in Toruń, 7 Gagarin St., 87-100 Toruń, Poland

**Keywords:** CNT oxidation, Ultrasonication, CNT toxicity, MSC cells, CHO cells

## Abstract

We discuss eight major challenges in the field of carbon nanomaterial toxicity. Generally, we pick up some of them, and the most important challenge is searching of the qualitative relationships between nanofactors and cytotoxicity. This is important since it can provide the possibility of conscious changes of carbon nanotubes cytotoxicity by manipulation with selected nanofactors. Therefore, the toxicity of a series of gradually oxidized carbon nanotubes is studied. We show, for the first time, that toxicity of those materials depends strongly on the ratio of acidic to basic group concentration—the higher is this ratio value, the more toxic are nanotubes. In this way, by changing this ratio, one can change toxicity. This correlation is more evident after ultrasonication, and it is connected with the accessibility of charged groups for interactions with proteins. Toxicity also depends on the ability of nanotubes for protein adsorption. We suggest that the changes in the protein composition of medium, especially lack of important growth factors, inhibit cell proliferation.

**Electronic supplementary material:**

The online version of this article (doi:10.1007/s12010-015-1607-1) contains supplementary material, which is available to authorized users.

## Introduction and the Aims of the Study

Nanotechnology and nanoscience expand rapidly, and this has led to the discovery and production of different new nanomaterials such as carbon nanotubes (CNT), being very important and widely used in different fields of science. Single-walled carbon nanotubes (SWCNT) are used in industrial and biomedical fields, such as electronic devices, wastewater treatment, and in possible drug delivery systems [1]. Owing to the wide use of CNT, they readily enter environment where they are more and more likely to be present and where they can associate with organic micropollutants due to strong sorption [2]. CNT can easily enter organisms by many pathways, such as inhalation [3] and/or injections and penetration [4, 5]. Thus, it is necessary to learn more about the possible toxicity of this new class of nanomaterials for occupational and environmental health. In this field, some tests show that CNT are nontoxic [6–8], especially at low concentrations; however, some authors claim that they are more toxic than, for example, asbestos [9]. The toxic effects of CNT on mammals and other species have been reported in many references, and the concern on them has increased significantly over the years. Thus, the subject of nanotube toxicity is relatively new and very important. The existing toxicological data are still fragmentary, and it is necessary to systematically summarize the advances in nanomaterial toxicity. Although much progress has been made in understanding the CNT toxicity in both in vivo and in vitro studies, some problems still remain unresolved. The CNT toxicity has been attributed to their length, dispersion, functionalization, concentration, exposure time, shape, defects, etc. [10–13]. However, the available data do not allow identifying specific CNT properties that are responsible for their cytotoxicity. It is well known that physicochemical modifications of CNT determine the state and stability of their suspension. Thus, the functionalization which increases solubility should lead to more toxic properties of nanotubes, among the others due to the higher cellular uptake [14–16].

Recently, in the study by Yan et al. [17], eight major challenges in the field of carbon nanomaterial toxicity were presented. It is well known that this toxicity can be changed by chemical modification since it changes the interactions of nanomaterials with cells. Thus, in this study, we tried to pick up some challenges mentioned in [17]. Authors conclude that there are no explicit relationships between the toxic responses and nanofactors. By the term “nanofactors,” the authors mean the characteristics of nanomaterials. Thus, in this study, we are looking for qualitative relationships between selected nanofactors and toxicity. Next challenge [17] is related to the controllable surface chemistry of nanomaterials. By the procedure of hydrothermal oxidation [18–20], we are able to add controllable number of oxygen groups on the surface of nanotubes; however, we can control mainly concentration of those groups. In this way, using the hydrothermal treatment procedure, being so-called “green chemistry” method, we also pick up challenge 3 [17]—we avoid organic solvents during modifications—and in this way, we reduce the unintended impurities. We also try to estimate some fundamentals of toxicity mechanism, being still unknown. Yan et al. [17] in challenge 5 pointed out that it is important to study comparatively the toxic responses of nanomaterial in comparison with the bulk material of identical chemical composition—this is why, in this study, we check the influence of sonification on toxic properties of nanotubes. This is also in agreement with the assay development workflow for toxicity screening of nanomaterials presented by Damoiseaux et al. [21]. Yan et al. [17] also pointed out (challenge 8) that there are many disagreements in the experimental nanotoxicology data; therefore, in our study we try to explain some of them.

Additionally, we can also add one next challenge to the mentioned above list. It is well known that nanotubes can be potentially applied in different areas of human activity. Toxicity of nanotubes can be used, for example, for microbial activity [22], and modification/coating of nanoparticles can reduce this toxicity, making possible application of nanotubes in nanomedicine. Thus, it will be interesting to get possibility of conscious changes of CNT cytotoxicity by manipulation with selected nanofactors. And, the results of our study show that this can be done. Moreover, at the same time, we try to understand the origin of the correlation between physicochemical properties and toxicity since it is very important for designing of safe nanomaterials [23]. Thus, the complex approach using different series of nanotubes and the performance of a series of systematic toxicity studies on different types of cells is necessary. Taking this into account, in the current paper, we show the preliminary cytotoxicity results of systematic and complex studies for the series of chemically modified nanotubes discussed previously [20].

## Materials and Methods

### Nanotubes

Commercial, high-purity, opened single-walled CNT from Nanostructured and Amorphous Materials (NanoAmor, Houston, TX, USA) were investigated (labeled as A0-o). They were hydrothermally oxidized in conc. 30 % H_2_O_2_ in the temperature range: 303–523 K (303, 453, 473, 493, and 523 K; the labeling of the samples is A0-o-*xxx* where *xxx* is the temperature in K). The detailed procedure, as well as some characteristics (the values of BET surface area, burn-offs, the values of enthalpy of immersion in benzene, thermal analysis results, and Raman spectra), were given previously [20]. Other characteristics discussed in this study, the concentration of surface acidic and basic groups, X-ray photoelectron spectroscopy (XPS) measurements results, and the pH of suspension values, are new and have not been published yet. The details of XPS and pH of suspension studies are provided in Supplementary Information.

In this study, the tested series of SWCNT with different surface chemical properties were dispersed by ultrasonication (3 h) in concentration of 1 mg/ml in 10 mmol/l PBS buffer pH = 7.4. Next, the adequate dose of the solution was added to cell culture medium to obtain final concentrations of 1, 10, and 50 μg ml^−1^. At this stage, the influence of ultrasonication (denoted as US) on toxicity was examined; i.e., tested CNT were dispersed ultrasonically for additional 5 min (in cell culture medium) or added to the wells without ultrasonication.

### In Vitro Studies

Chinese hamster ovary (CHO) cells were obtained from Sigma-Aldrich. Cells were grown in F-12 medium containing 10 % fetal bovine serum (FBS) at 310 K in a CO_2_ incubator with 5 % of CO_2_. A volume of 25 μl containing approximately 5 × 10^5^ cells was seeded to each well of a 12-well plate 24 h before the experiment was started. Human mesenchymal stem cells (MSC) from umbilical cord were purchased from PromoCell. Cells were grown in MSC growth medium with 10 % SupplementMix (both from PromoCell) at 310 K in a CO_2_ incubator with 5 % of CO_2_. A volume of 25 μl containing approximately 5 × 10^5^ cells was seeded to each well of a 12-well plate 24 h before the experiment was started.

CNT samples were added to the growing CHO and MSC cells in concentrations of 1, 10, and 50 μg ml^−1^, respectively, and incubated for the next 24 h. Subsequently, the 3-(4,5-dimethylthiazol-2-yl)-2,5-diphenyltetrazolium bromide (MTT) and LDH activity tests were performed in triplicate. The detailed description of cytotoxicity tests is given in Supplementary Information.

### Protein Adsorption Analysis

Two hundred microliters of 1 mg/ml A0-o-493 tubes (with or without sonication, respectively) was added to 800 μl of 5 % FBS in F-12 medium and incubated for 12 h at 310 K. After the designated time period, the sample was centrifuged (25,000 *g*, 5 min) and the absorbance in supernatant was measured at 280 nm. Electrophoresis in denaturing conditions: polyacrylamide gel electrophoresis in denaturing conditions (sodium dodecyl sulfate-polyacrylamide gel electrophoresis (SDS-PAGE)) was performed according to the Ogita and Markert method [24]. For protein separation, 4 % stacking gel and 7.5 % running gel were used. Electrophoresis was run at constant voltage of 120 V in electrode buffer: 25 mmol/l Tris-glycine, pH 8.3, containing 1 % SDS. Analyzed samples (5 % FBS in F-12 medium with 0.5 mg CNT with or without sonication, respectively, incubated for 12 h at 310 K) were denaturated for 5 min at 370 K in reducing buffer containing 4 % SDS and 10 % β-mercaptoethanol prior to loading onto the gel wells. After separation, proteins were visualized with 0.1 % Coomassie Brilliant Blue R-250 staining.

### Statistical Analysis

All experiments were repeated at least three times, and qualitatively identical results were obtained and the average data are reported.

## Results and Discussion

**Fig. 1:**
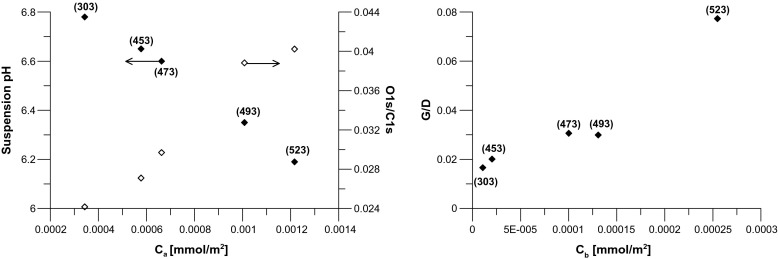
The correlations between the concentration of surface acidic (*c*_a_) and basic (*c*_b_) groups of studied nanotubes and selected carbon characteristics (see Table 1S)

Table 1S and Fig. 1S (Supplementary Information) collect new characteristics of studied nanotubes and the examples of XPS spectra. The analysis of the results leads to the conclusion about the presence of typical forms of carbon and oxygen functionalities observed in carbon materials [19, 25]. Thus, the analysis of C1s spectra leads to the conclusion that for all studied nanotubes, the following forms of carbon are present: graphitic (marked as A; ca. 284.2 eV); alcoholic, aliphatic ether, or other sp^3^ (B; ca. 285.2 eV); phenolic and/or aromatic ethers (C; ca. 286.4 eV); carbonyl or quinone (D; ca. 286.6 eV); carboxyls and esters (E; ca. 289.0 eV); and *π* → *π* * (F; ca. 290.4 eV). The analysis of O1s peaks shows the presence of molecular oxygen (A; ca. 530.2 eV); oxygen from carbonyl functionalities (B, ca. 531.6 eV); oxygen singly bonded to carbon in phenols, ethers, or aromatic ring (C; ca. 534.0 eV); and occluded CO_2_ or H_2_O (D; ca. 536.0 eV). The comparison of calculated peak areas leads to the conclusion that the number of oxygen groups on initial nanotube surface is not very high. The progressive change of modification conditions leads mainly to the creation of surface carboxyl and carbonyl groups. For all studied samples, the ratio of atomic percentage content determined from the XPS spectra (denoted as O1s/C1s) was calculated (see Table 1S). One can see that the application of hydrothermal treatment procedure leads to progressive burn-off of studied samples and, at the same time, to the progressive rise in surface polarity (see, e.g., [20]). Some correlations, obtained from XPS results, are collected in Fig. 1. One can observe that studied series of nanotubes have similar properties to activated carbons and carbon blacks; i.e., with the rise in concentration of surface acidic groups, the ratio of O1s/C1s XPS peaks increases (see [19, 25] and references therein). Obviously, with the rise in concentration of acidic groups, we observe the decrease in the value of pH of suspension. It can be mentioned here that in the cell culture medium with nanotubes, the pH level (7.4) remains stable.

It is interesting that we do not observe the correlation between the concentrations of acidic and basic groups, reported often for activated carbons [26]. It was concluded that for different activated carbons, the rise in surface acidity is caused by the simultaneous decrease in surface basicity. If we assume that the basic carbon centers are of Lewis’s type, the creation of acidic groups occurs by a reaction between oxidant and those basic functionalities. Contrary, in the case of studied nanotubes, we observe that with the rise in acidity, simultaneous rise in basicity occurs. Moreover, as it is shown in Fig. 1, the rise in basicity is related to the rise in the *G*/*D* ratio. As it was concluded previously [20] for studied series, with the rise in burn-off (i.e., from A0-o up to A0-o-523) as well as with the rise in the number of acidic groups, we observe simultaneous decrease in the content of amorphous carbon. In our opinion, it means that the rise in basicity is the result of the percentage increase in the content of graphitic nanotube walls since with the progress in surface oxidation, amorphous carbon is removed. In this way, for studied nanotubes, increase in surface acidity is accompanied by a simultaneous increase in surface basicity.

There are some evidences that charge of the surface of nanoparticle may be also relevant for toxicity. In fact, both positively [27] and negatively charged nanomaterials have been found to be more injurious to cells than noncharged ones. In general, it is believed that cationic surfaces are more toxic than anionic surfaces due to the affinity of cationic particles to the negative phospholipid head groups of protein domains on cell membranes. In vitro studies of Bhattacharjee et al. [28] show that cationic nanoparticles exhibit toxicity on two different human and rat cell lines. However, in some studies, negatively charged nanoparticles were more reactive than positively charged ones [29, 30]. It is therefore possible that the effect of charge depends on the type of nanomaterial and experimental conditions, including cell type and tissue or organism tested. Among those experimental conditions, Wick et al. [31] mentioned CNT dispersion as another important property influencing cytotoxicity.

According to above information, following the currently proposed workflow required for nanomaterial toxicity screening [21] in this study, we test the series of gradually oxidized nanotubes against their toxic influence on two different cell lines. Chinese hamster ovary (CHO) cells represent an animal, stable line of differentiated, specialized cells, while mesenchymal stem cells (MSC) are undifferentiated cells of human origin. We have applied two different tests to determine the probable mechanism of cell damage. MTT test detects early cytotoxic events based on the decrease in activity of mitochondrial enzymes, whereas LDH leakage is triggered by the cell membrane damage and release of the enzyme into the culture medium.

**Fig. 2:**
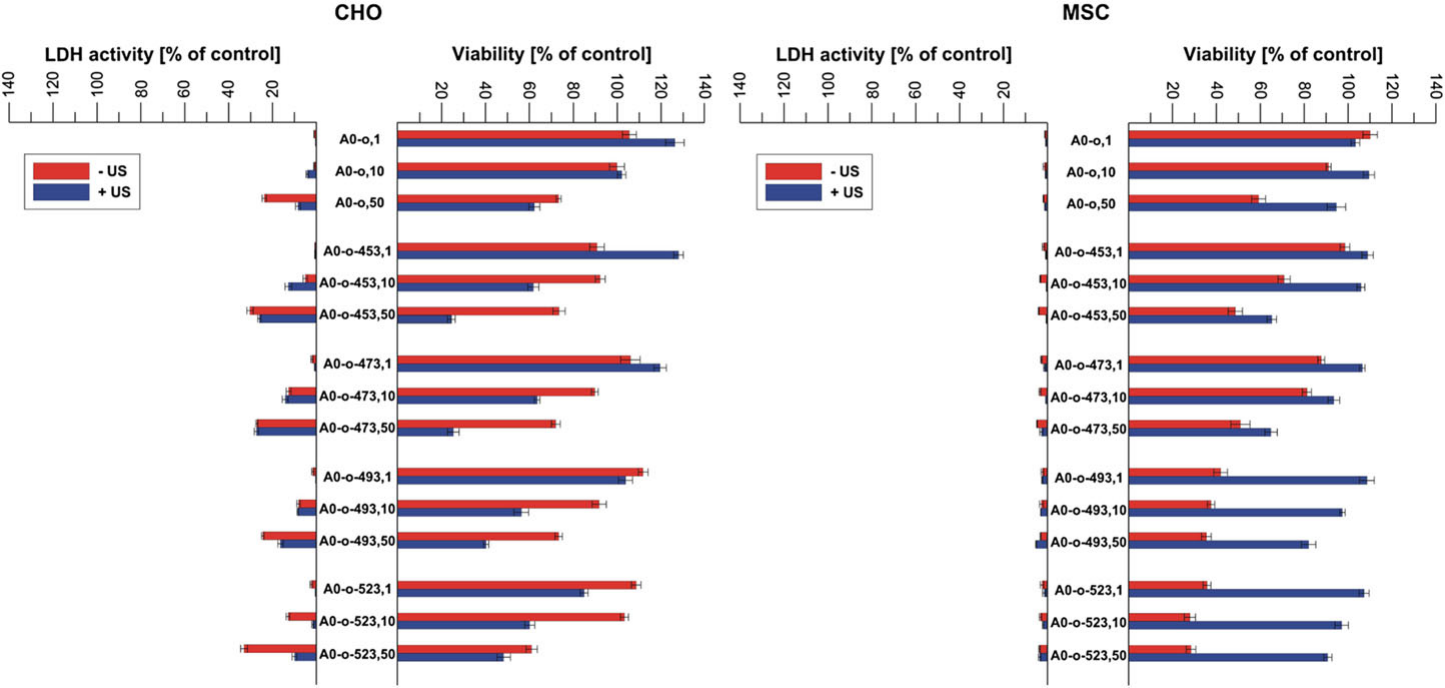
The influence of ultrasonic treatment on the toxicity of series of nanotubes. The results show the viability of CHO and MSC cells determined by MTT tests and LDH activity. Numbers *1*, *10*, and *50* denote the dose in microgram per milliliter, and *US* denotes ultrasonication

Figure 2 shows the effect of ultrasonic treatment on toxicity of nanotubes. The results suggest that strongly oxidized CNT without ultrasonication (US) are toxic for quickly dividing, undifferentiated cells as MSC cells. The US treatment reduces this effect completely, even for as high concentration as 50 μg/ml. However, for regular, differentiated, and immortalized cells as CHO, the toxicity is observable only for the highest tested concentrations after US treatment. According to the results of Wick et al. [31], suspended nanotubes are less toxic than bundled. However, from the results of our study, one can conclude that we do not observe the general regularity; i.e., the application of ultrasounds can increase or decrease toxicity of nanotubes; however, it depends not only on the type of a sample (i.e., the surface chemical nature) but also on the type of cells applied for tests. As seen for MSC cells, the CNT cytotoxicity is connected with intracellular effects (changes in mitochondrial activity determined by MTT test) before any cell membrane damage occurred. Summing up, undifferentiated and differentiated cells respond differently to CNT-induced cytotoxicity.

These results are in excellent agreement with those reported by Jan et al. [32]. Authors suggested that undifferentiated cells are less sensitive and vulnerable for cytotoxic influence of nanomaterials. It is worth to note that in the case of MSC cells, the cytotoxic effect is connected rather with cytostatic activity than with cell death, due to the fact of the very low LDH activity through all experiments.

**Fig. 3:**
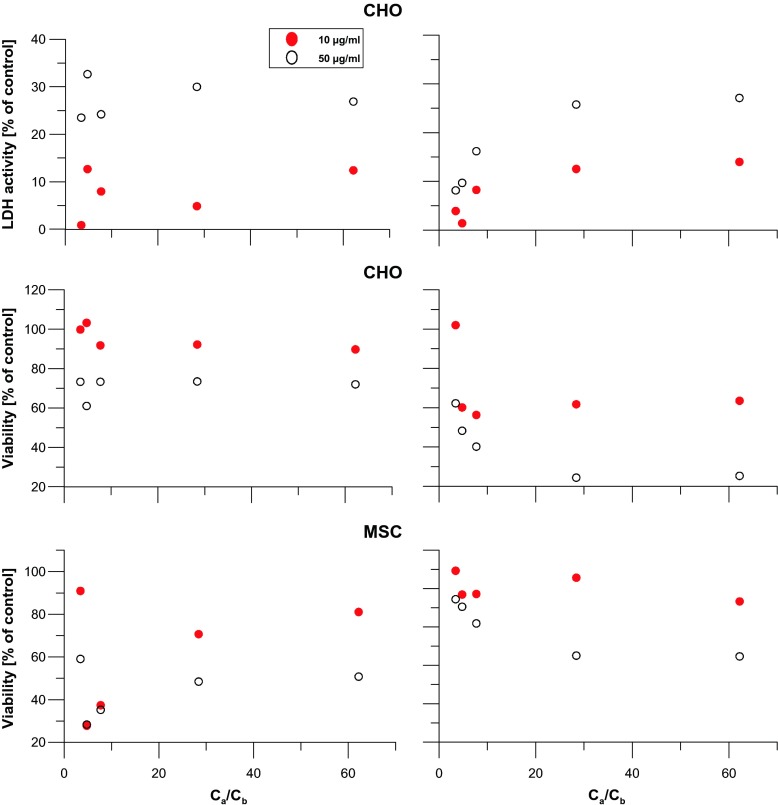
The influence of ultrasonic treatment on the general correlations between the viability and LDH activity of CHO and MSC cells, plotted as a function of the acidic to basic surface group concentration ratio (*c*_a_/*c*_b_) for two different CNT concentrations. *Left panel—*tests without US, *right panel—*with application of US

**Fig. 4:**
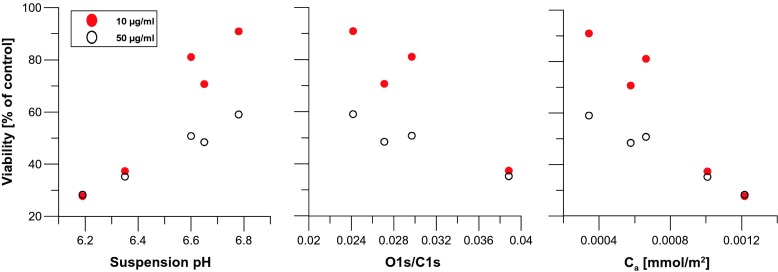
MSC cell viability determined by MTT tests as a function of carbon nanotube characteristics (Table 1S). Tests were performed for two different CNT concentrations without US

Interestingly, decrease in viability of MSC cells together with very low LDH activity suggests distinct biological response of undifferentiated cells. Thus, we tried to find the general correlations between LDH activity as well as cell viability (from MTT assay) and nanofactors collected in Table 1S, as shown in Fig. 3. For CHO cells, the ratio of concentration of surface acidic and basic functionalities (*c*_a_/*c*_b_) does not influence the LDH activity. As one could expect, the activity depends only on CNT concentration. Additional US treatment causes decreasing LDH activity especially for low *c*_a_/*c*_b_ values. The results from MTT assay for samples without US treatment show similarly only the concentration dependence, remaining constant for all *c*_a_/*c*_b_ values. However, the US treatment causes, in this case, drastic toxicity effect and viability decreases down to ca. 20 % for high *c*_a_/*c*_b_ ratios. Contrary, for MSC cells, the US treatment causes an increase in viability, especially for low *c*_a_/*c*_b_ values. The analysis of observed correlations shows that *c*_a_/*c*_b_ ratio is an important factor changing the toxicity. However, as one can have direct influence on number of acidic groups on the surface of CNT and the basicity remains a derivative factor, consideration of the surface acidity is of decisive importance for understanding the cytotoxicity. Figure 4 summarizes the most important correlations between parameters characterizing acidity of carbon surface and their cytotoxic properties. The results are presented for MSC cell viability, calculated from the MTT test. It is clear that the rise in surface acidity increases the toxicity effect.

**Fig. 5:**
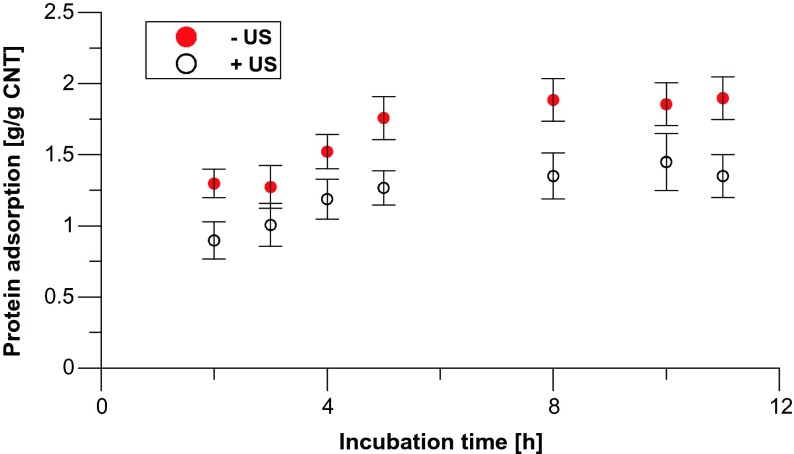
Total protein adsorption on CNT plotted as the function of time

**Fig. 6:**
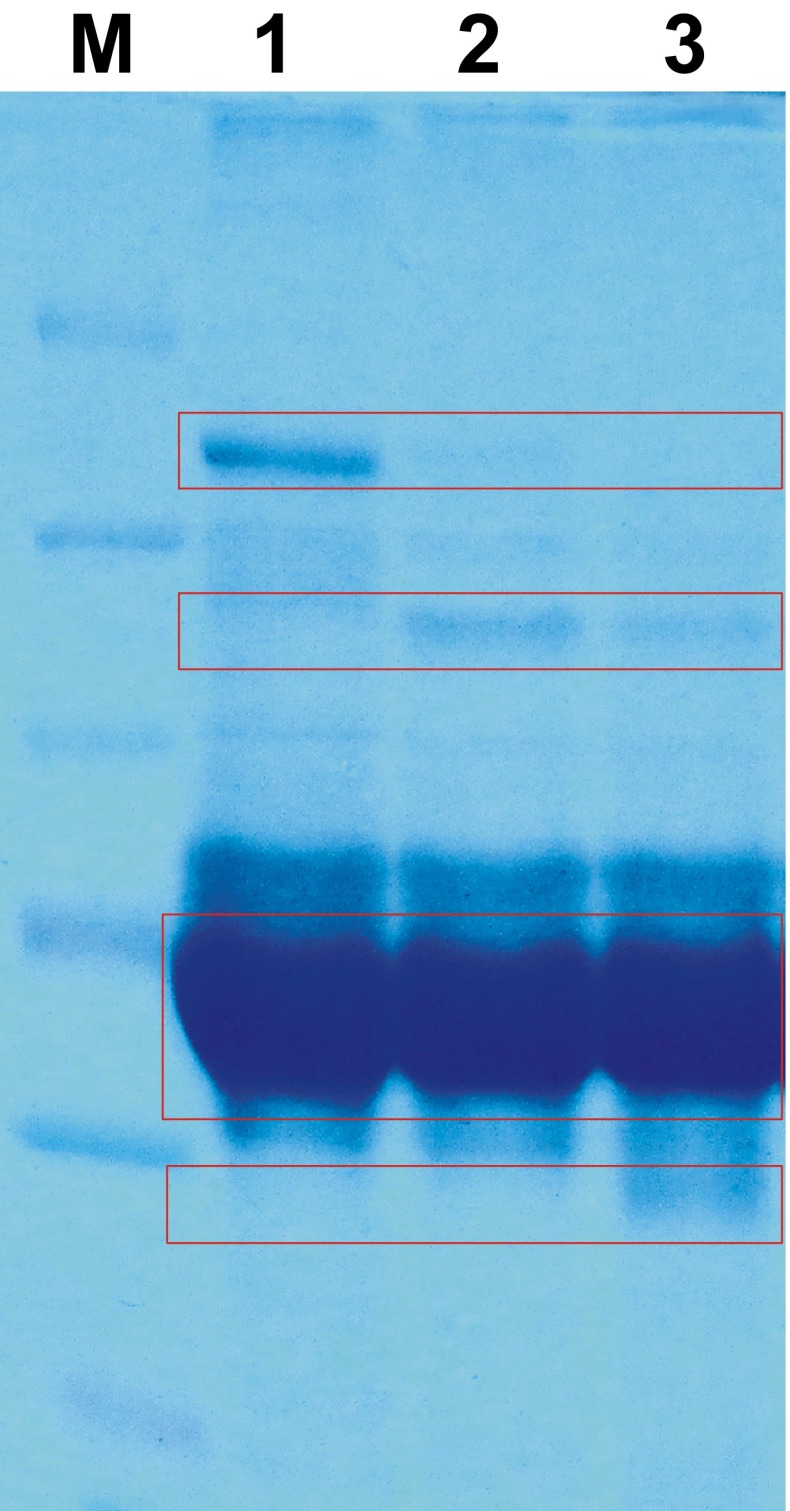
SDS-PAGE electrophoretic separation. *M* molecular weight marker, *lane 1* 5 % FBS in F-12 medium after 12-h incubation, *lane 2* 5 % FBS in F-12 medium after 12-h incubation with nondispersed CNT, *lane 3* 5 % FBS in F-12 medium after 12-h incubation with ultrasonically dispersed CNT. *Red frames* indicate the main differences between samples

In order to shed some light to explanation of the mechanism of toxicity, we decided to determine the protein adsorption capacity of nanotubes A0-o-493, since they show large differences in toxic properties if they are applied with or without US treatment (see Fig. 2). Figure 5 presents the differences in total protein adsorption (from 5 % FBS in F-12 medium) on studied nanotubes (with or without ultrasonic dispersion, respectively) during 12-h incubation. Protein adsorption (under conditions without ultrasonic treatment) rises with time of the process up to ca. 1.8 g/g CNT reaching the maximum after ca. 8 h. Interestingly, almost twice reduction of adsorption capacity is observed as a result of 5-min ultrasonic treatment at the beginning of the process. The results mean that nanotubes in the solution are able to form bundles with pores accessible for (and able to adsorb) proteins. Additional US dispersion in the protein solution leads to partial destroying of these structures [31] and decrease in adsorption capacity. In parallel, ultrasonic dispersion of CNT increases accessibility of charged groups for interactions with proteins. These groups may facilitate the binding of physiologically charged proteins outside the cell as proved by SDS-PAGE electrophoresis. The electrophoretic separation of cell culture medium proteins remaining after 12-h incubation with nanotubes explains the nature of CNT-protein interactions, as presented in Fig. 6.

As it was shown above, the CNT properties determine their toxicity for cell cultures; i.e., nanotubes dispersed by ultrasounds seem to be more toxic for CHO cells and apparently less toxic for MSC. When considering cell physiology, sensitivity of MSC and CHO cells is obviously different. Physiological response of MSC to signals from the extracellular space provides the maintenance of high proliferation rate and their self-renewal. Moreover, MSC cells have better detoxification mechanisms and ability to adapt for changes in extracellular environment [32]. We show that only large, nondispersed nanotube aggregates can decrease the proliferation rate of MSC and/or activate the cell death processes. As the MSC-qualified medium consists of different regulatory proteins and growth factors and nondispersed CNT adsorbed ca. two times more proteins than sonicated ones, we suggest that changes in the protein composition of medium, especially lack of important growth factors, inhibit cell proliferation. In the case of CHO cells, nondispersed CNT are less toxic than dispersed ones. Their presence in extracellular environment is recognized as a pathological signal, and its strength is proportional to CNT amount in the culture medium. Another possible explanation for CHO cells is that dispersed by US CNT can easily enter the cell and therefore are more toxic. However, the CNT penetration into the cell is still the problem that requires additional studies. [33] Large surface of CNT enables the strong sorption of growth factors and other proteins important for cell growth and proliferation. The interactions between nanotube surface and proteins of culture medium seem to be specific and selective (as proved by SDS-PAGE electrophoresis). Considering amphoteric nature of proteins, we suggest that accessibility of charged groups on CNT surface is important for the interactions. These groups may facilitate the binding of physiologically charged proteins outside the cell and/or interactions with the cell membrane.

The number of acidic and basic groups on the CNT surface is another important property of them. The *c*_a_/*c*_b_ ratio affects toxicity of CNT—the higher is the ratio value, the more toxic are nanotubes to CHO and MSC cells. This correlation is more evident with US application, and it is connected with the accessibility of charged groups for interactions as it was explained above.

## Conclusions and Perspectives

In this study, pick up some challenges formed by Yan et al. [17], we study the materials with systematically changed surface properties through gradual oxidation of CNT. Thus, we prove that CNT cytotoxicity depends strongly on surface properties and dispersion of the tested materials. However, various types of cells respond differently. Cytotoxicity of tested materials for CHO cells is low (only the highest CNT concentration significantly decreases cell viability) and results mainly from ultrasonic dispersion of CNT. Distinct CNT cytotoxicity for MSC cells suggests different biological response of undifferentiated cells and is apparently correlated with *c*_a_/*c*_b_ ratio with simultaneously limited influence of ultrasonic dispersion. Up to our knowledge, this is the first paper showing quantitative relationships between nanofactors and cytotoxicity. Knowing this, by the change of *c*_a_/*c*_b_ ratio, one can change cytotoxicity.

Our results definitively indicate that CNT-induced cytotoxicity is a complex issue, evaluation of which requires systematic and versatile studies carefully considering CNT properties, cells chosen as an in vitro model, and applied toxicity tests.

## Electronic supplementary material

ESM 1 (DOC 166 kb)
