# Supplementary material for: Conscious Changes of Carbon Nanotubes Cytotoxicity by Manipulation with Selected Nanofactors
Source: Appl Biochem Biotechnol. 2015 Apr 18;176(3):730–41. doi: 10.1007/s12010-015-1607-1 (PMC4500856; doi:10.1007/s12010-015-1607-1)
Supplement: Supplementary file 1 — (DOC 166 kb) [file 12010_2015_1607_MOESM1_ESM.doc]

**Supplementary Information**

**Table 1S.** The characteristics of studied series of A0-o nanotubes

| **Sample labeling** | **c_a_ 10^4^ _(mmole/m_^2^)** | **c_b_ 10^4^**  **_(mmole/ m_^2^)** | **pH_susp_** | **Raman G/D 10^3^** | **XPS**  **O_1s_/C_1s_ 10^3^** |
| --- | --- | --- | --- | --- | --- |
| A0 - o | 3.43 | 1.00 | 6.78 | 30.6 | 24.17 |
| A0 - o - 453 | 5.78 | 0.20 | 6.65 | 20.1 | 27.11 |
| A0 - o - 473 | 6.63 | 0.11 | 6.60 | 16.6 | 29.69 |
| A0 - o - 493 | 10.1 | 1.31 | 6.35 | 29.9 | 38.83 |
| A0 - o - 523 | 12.2 | 2.55 | 6.19 | 77.3 | 40.23 |

*c_a_, c_b_* - the concentration of surface acidic and basic groups, respectively, determined using the method of Boehm (Boehm, H. P., 1994, Some Aspects of the Surface Chemistry of Carbon Blacks and Other Carbons, Carbon 32, 759 - 769), *pH_susp_* - pH level of suspension in 0.1 mole/l NaNO_3_, *G/D,* O_1s_/C_1s_ - the ratio of graphitic to disordered carbon intensity determined from Raman spectra, or the ratio of oxygen/carbon states from XPS spectra, respectively


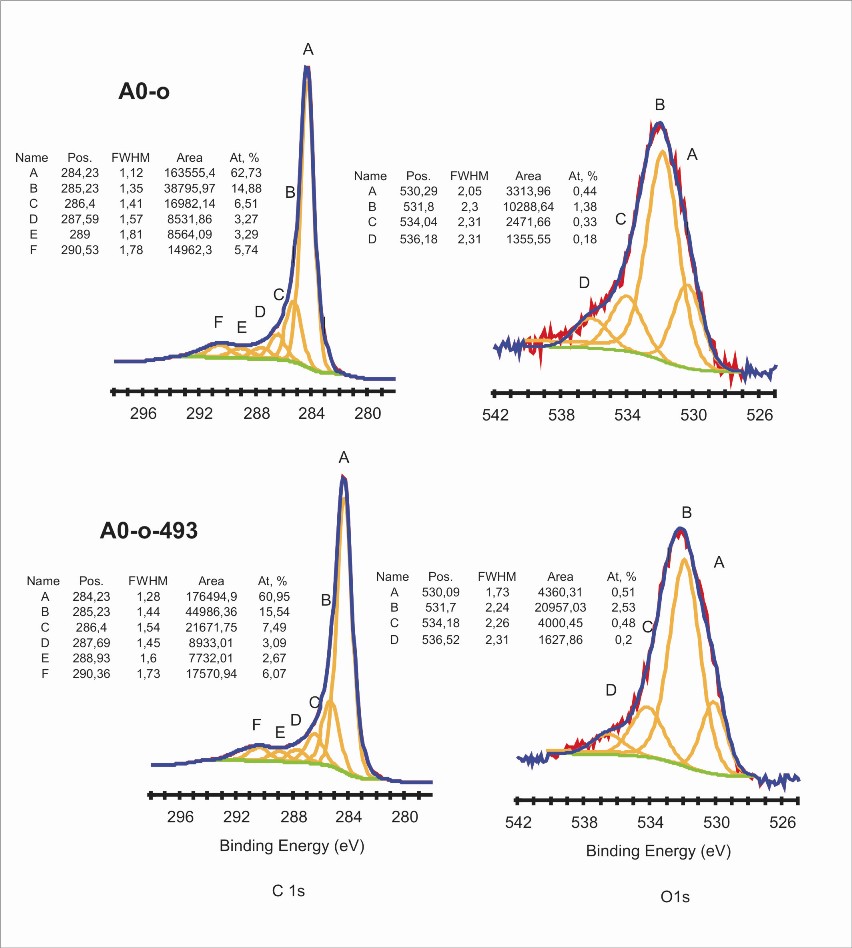


Figure 1S. The XPS spectra of selected nanotubes A0-o and A0-o-493.

*Spectroscopic and pH of suspension analyses*

XPS measurements were performed using a VG Scientific photoelectron spectrometer ESCALAB-210 using Al Ka radiation (1486.6 eV) from an X-ray source operating at 15 kV and 20 mA. Survey spectra were recorded for all the samples in the energy range from 0 to 1350 eV with 0.4 eV step. High-resolution spectra were recorded with 0.1 eV step, 100 ms dwell time and 25 eV pass energy. Ninety degrees take-off angle was used in all measurements. The curve fitting was performed using the AVANTAGE software provided by Thermo Electron, which describes each component of the complex envelope as a Gaussian–Lorentzian sum function; a constant 0.3(±0.05) G/L ratio was used The background was fitted using nonlinear Shirley model. Scofield sensitivity factors and measured transmission function were used for quantification.

The values of suspension pH were measured according to the procedure described previously (Pacholczyk et al., 2011).

*Cytotoxicity tests*

The MTT assay was conducted as follows: the MTT solution in a concentration of 1 mg ml^-1^ in F-12 medium was added to the wells with CHO or MSC cells after 24 h of cells incubation with different concentrations of nanomaterials. After 15 min incubation the solution was discarded and formazan crystals were dissolved in DMSO. The absorbance at 570 nm (with background absorbance at 750 nm) was measured spectrophotometrically.

LDH activity can be determined in culture medium by decrease in reduced form of NAD (NADH - reduced β-nicotinamide adenine dinucleotide). The measurements were performed spectrophotometrically at 340 nm. The decrease in the amount of NADH directly correlates to the increase in the number of damaged cells. For LDH activity tests culture medium was collected from the wells after 24 h of cells incubation with different concentrations of nanomaterials. LDH activity tests were performed as follows: 100 μl of NADH (1.25 mg/ml) and 100 μl of sodium pyruvate (2.5 mg/ml) were added to 600 μl of culture medium. The absorbance at 340 nm was measured spectrophotometrically. The number of living and damaged cells was compared to the control sample considering the positive control (100% damaged cells).
